# IMPACT OF ADAPTED DANCE ON MOOD AND PHYSICAL FUNCTION AMONG ALZHEIMER’S DISEASE ASSISTED LIVING RESIDENTS

**DOI:** 10.1093/geroni/igz038.2784

**Published:** 2019-11-08

**Authors:** Crystal Bennett

**Affiliations:** University of West Florida, Pensacola, Florida, United States

## Abstract

Neuropsychiatric secondary symptoms and altered physical function are prevalent among persons with Alzheimer’s Disease and related dementia disorders (ADRD) which increase healthcare costs and caregiver burden. Adapted dance is a promising intervention that may improve these symptoms and physical function. The purpose of this study is to test whether 12 weeks of adapted dance (60 min 2x/week) improves agitation, physical function, and reduces caregiver burden. An experimental crossover design will be used. ADRD residents with a Montreal Cognitive Assessment score between 6-26, Timed up and go of <20 seconds, Cohen-Mansfield Agitation Inventory Score (CMAI) of >15, and do not use oxygen or assistive device will be eligible to participate. Outcomes will be assessed at baseline and every 4 weeks during each study arm for 24 weeks. Measures include CMAI and the Neuropsychiatric Inventory-Clinician Scale for agitation; Short Physical Performance Battery for physical function; and Zarit Burden Interview for caregiver burden.

